# Prognostic Factors of Pulmonary Metastasectomy for Oligometastatic Hepatocellular Carcinoma Spread to the Lungs

**DOI:** 10.3390/jcm13144241

**Published:** 2024-07-20

**Authors:** Bohyun Kim, Mi Hyoung Moon, Seok Whan Moon

**Affiliations:** 1 Department of Radiology, Seoul St. Mary’s Hospital, College of Medicine, The Catholic University of Korea, Seoul 06591, Republic of Korea; kbh@cathoilc.ac.kr; 2 Department of Thoracic and Cardiovascular Surgery, Seoul St. Mary’s Hospital, College of Medicine, The Catholic University of Korea, Seoul 06591, Republic of Korea

**Keywords:** pulmonary metastasis, hepatocellular carcinoma, metastasectomy, prognosis, survival

## Abstract

**Background/Objectives**: Pulmonary metastasis is the most prevalent type of extrahepatic hepatocellular carcinoma (HCC) metastasis. International guidelines recommend systemic treatment for patients with HCC having pulmonary metastases. However, the role of pulmonary metastasectomy (PM) remains relatively unexplored. Therefore, we assessed the survival outcomes and the factors influencing them in patients who underwent PM for metastatic HCC. **Methods**: Clinical data were collected from patients with HCC who underwent PM for metastasis at a single tertiary center between January 2004 and December 2022. Recurrence-free survival and overall survival were assessed using Kaplan–Meier curves. The Cox proportional hazards model was used to identify factors associated with survival outcomes. **Results**: Overall, 63 patients underwent PM with a median follow-up time of 84.0 months. The cumulative survival rates after the initial PM at 1, 2, and 5 years were 79.1%, 63.9%, and 35.6%, respectively. In multivariate analysis, early intrathoracic recurrence <6 months, number and size of metastases, resection margin status, and PM bilaterality were significantly associated with overall survival. A larger size of the primary HCC, increased number of repeated PM, and frequent lobectomy were more common in patients with early (<6 months) recurrence after PM than in those without early recurrence. **Conclusions**: PM in patients with metastatic HCC may provide acceptable survival outcomes for those with smaller, unilateral lung metastases that can be resected with generous surgical margins. However, early recurrence with reduced overall survival is likely in patients with a larger-size initial HCC after prior PM and lobectomy.

## 1. Introduction

Hepatocellular carcinoma (HCC) is one of the most fatal cancers worldwide. According to recent statistics, the 5-year survival rate in advanced stages remains <20% despite oncological advancements [1]. Furthermore, the recurrence rate is high. A nationwide study by the Liver Cancer Study Group in Japan found that 34.4% of patients experienced intrahepatic or extrahepatic recurrence within 2 years of diagnosis. The most typical sites of extrahepatic recurrence were the lungs, the bones, and lymph nodes. Patients with pulmonary metastases have an extremely dismal prognosis; median overall survival (OS) is 3 months according to Surveillance, Epidemiology, and End Results (SEER) data compared with 19 months in patients without pulmonary metastases. The critical issues for these patients include determining the timing of lung metastasis and identifying effective treatments. SEER data indicate that larger initial HCC sizes (>7 cm in diameter), concurrent bone or brain metastases, and poor differentiation increase the risk of pulmonary metastases [2]. Another critical concern is identifying the most beneficial treatment for pulmonary metastases.

Pulmonary metastasectomies (PMs) have been typically performed for diagnostic and therapeutic purposes for several decades. Since the 1970s, when its surgical role was first described, PM has gradually increased and become a standard procedure in clinical practice. However, evidence for its effectiveness is surprisingly weak, particularly compared with other procedures [3]. The role of PM in metastatic HCC has been largely unrecognized. Current international guidelines commonly recommend systemic treatment for metastatic HCCs, regardless of their location [4,5,6,7]. Specifically, the guidelines support the combination of atezolizumab and bevacizumab or durvalumab and tremelimumab as a first-line systemic treatment for patients with extrahepatic disease. While radiation therapy is used in patients with oligometastatic HCC in various parts of the body, including the lungs, this practice has not been incorporated into guidelines [8]. However, a recent report suggested that PM may offer a promising approach for managing isolated pulmonary metastases. These findings indicated that PM can significantly enhance the 1-year survival rate from 20% to 42% [9]. Additionally, in recurrent HCC after liver transplantation, radical PM provides better survival rates than those without surgical treatment [10], highlighting the potential role of PM in the management of metastatic HCCs.

Conducting reliable and accurate studies on metastatic disease is challenging, whether in prospective or retrospective settings. The clinical heterogeneity and characteristics of the populations make ongoing randomized controlled trials (RCTs) on the clinical efficacy and survival benefits of PM scarce. Notably, only a few exist for diseases such as osteosarcoma, other bone tumors, and breast cancer [11]. Currently, there are no prospective RCTs evaluating PM for HCC, and most previous retrospective studies involved relatively small patient cohorts, limiting the generalizability and interpretability of their findings.

The decision to perform PM depends on various factors, including patient medical fitness, underlying liver function, lesion resectability, and physician preference. Selection bias is inevitable because, generally, patients undergoing PM have conditions tolerable for surgery. Consequently, defining a “comparable” control group is difficult, and whether a particular variable is prognostic or predictive is often unclear. Additionally, the follow-up duration varies, and the exact mechanism by which PM increases survival remains ambiguous [12].

Therefore, in this study, we aimed to assess the survival outcomes of patients with HCCs who underwent PM and identify prognostic factors, including the presence of viable HCC in the liver, while considering several clinical scenarios within a limited number of patients.

## 2. Materials and Methods

### 2.1. Datasets

In this retrospective study, we targeted patients with pulmonary metastasis from HCC who underwent PM between 1 January 2004 and 31 December 2022. Notably, all patient data were extracted anonymously from the Catholic Medical Center clinical data warehouse at Seoul St. Mary’s Hospital. We collected the following information: (1) diagnoses and comorbidities based on International Classification of Diseases (ICD-10) codes; (2) medication use; (3) laboratory data; (4) imaging data and readings of computed tomography (CT)/magnetic resonance imaging (MRI); (5) pathology reports; and (6) hospitalization summaries, operative records, and other outpatient electronic medical records (EMRs) that enable longitudinal follow-up. This provided us with basic demographic and diagnostic data, surgical events (operative date, extent of surgery, clinical course after surgery, and number of masses), admission and treatment details, and follow-up records of recurrence and survival.

**Figure 1:**
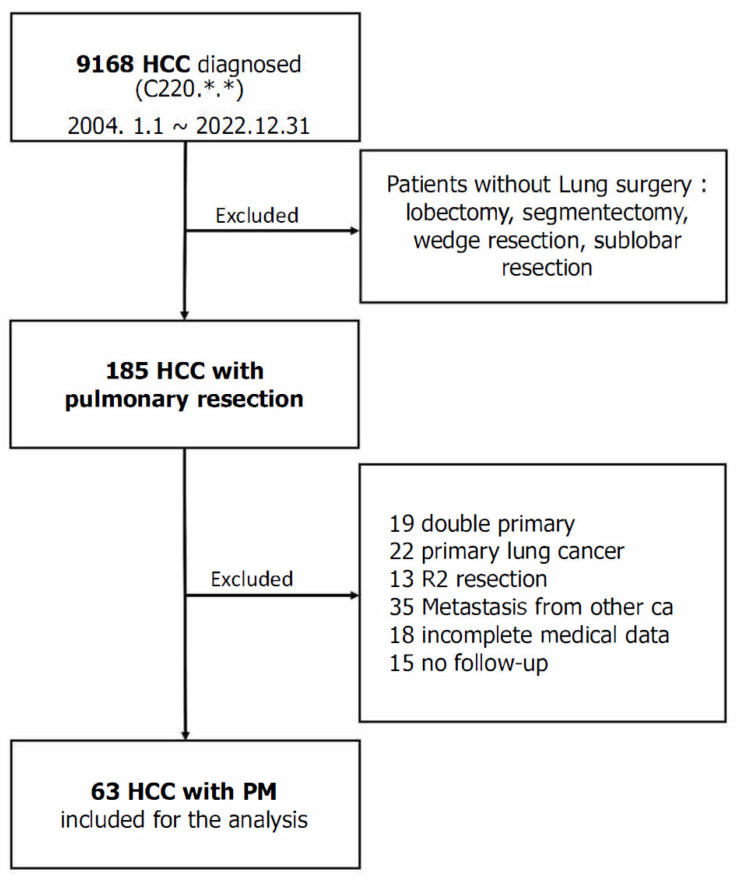
Flowchart of the patient enrollment process for this study. *.* means any numbers between 0 to 9.

The study population included patients who were assigned the ICD code C220 for HCC during the study period and those who were prescribed a thoracic surgery code (wedge resection, sublobar resection, segmentectomy, lung resection, lobectomy, or thoracoscopic exploration) during hospitalization. Patients were excluded based on the following criteria: (1) the pathology of the lung mass was not metastatic HCC, (2) incomplete medical data, (3) no information about follow-up status, and (4) diagnostic purpose of surgery (R2 resection). Notably, 185 of the 9168 patients with HCC underwent any lung surgery, and 63 were included in the dataset (Figure 1).

Regarding treatment history, the initial approaches for managing HCC include arterially directed therapies (transarterial embolization (TAE) or chemoembolization (TACE)), chemotherapy, and surgical options (including hepatectomy or liver transplantation for some patients). Additionally, any recurrence before the onset of the initial pulmonary metastasis and treatments administered before and after the first PM was documented. For PM, any scheduled two-stage operation to address bilateral pulmonary metastases was considered a single surgical event, provided the planned surgeries were performed within reasonably set timeframes.

Notably, all patients who underwent PM in our hospital met the following criteria: (1) pulmonary metastasis was regarded as resectable based on CT scans; (2) tolerable patient condition for general anesthesia with sufficient hepatic and pulmonary reserve; (3) metastatic disease was limited to the lungs, or at least other systemic metastases were in a remission state or controllable; and (4) locoregional control of HCC was obtained or obtainable. We included patients with primary HCC and synchronous metastases, as well as those with a history of controlled recurrence other than that in the liver or lung.

### 2.2. Variable Definitions

The definitions of the critical clinical parameters used in this study are summarized in Table 1. The different time points utilized in this study were defined as follows: T0 represents the time when pulmonary metastasis was initially suspected on chest CT surveillance, T1 denotes the time when PM was first performed, T2 signifies the time of the first intrathoracic recurrence following PM, and T3 represents the time of the last follow-up or death.

This retrospective study was conducted following the Declaration of Helsinki (revised in 2013) and was approved by the Institutional Review Board of the Catholic Medical Center (IRB No. KC22WIDI0477). The requirement for written informed consent was waived because all extracted data were anonymized.

### 2.3. Statistical Analysis

In this study, we primarily aimed to identify the risk factors associated with OS after the first PM for HCC. Study participants were grouped based on their pulmonary status after initial PM (recurrence vs. non-recurrence). We expressed patient characteristics as standard statistical parameters, including mean ± standard deviation (SD) or median values (continuous variables) and frequencies or percentages (categorical variables). Distributions of continuous variables were analyzed using Student’s *t*-test or the Mann–Whitney *U*-test, depending on the Shapiro–Wilk normality test results. Categorical variables were compared using the chi-squared or Fisher’s exact tests. The Kaplan–Meier method was used to assess recurrence-free survival, and the log-rank test was used to compare survival curves stratified by group.

The sample size for this study was determined using survival data from patients with HCC and pulmonary metastasis. The initial research question addressed the relationship between viable HCC in the liver and prognosis following the first PM. Eight of the nine patients with viable (progressive or partial response) HCC and seven out of eighteen patients with nonviable HCC (complete response group) died. Based on calculations by Chow et al., a sample size of 29 per group was deemed to be the minimal number to address the research question, and we proceeded with our analysis [13,14].

We used Cox proportional hazards (PH) models to investigate the factors associated with OS after the initial PM. Notably, several multivariate models were explored based on the Akaike information criteria (AIC) to select the best fit for the data. All analytics were driven by conventional freeware (R v4.3.3; the R Project for Statistical Computing, Vienna, Austria), with statistical significance set at *p* < 0.05.

## 3. Results

### 3.1. Clinical Presentations and Patient Characteristics

**Figure 2:**
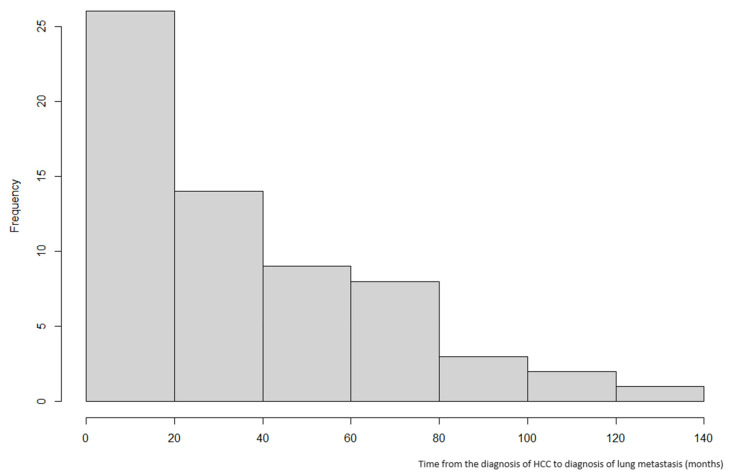
Time from diagnosis of initial HCC to identifying pulmonary metastasis.

The clinical characteristics of the 63 patients included in this study are summarized in Table 2. The mean age at the first PM was 55 years old, and 45 patients (71.4%) were male. The median follow-up time after the first PM was 28.0 months (interquartile range (IQR): 13.0–60.5 months), and the mean interval from the initial HCC diagnosis to the initiation of treatment was 16.6 days. Among these patients, 19 (30.2%) underwent surgery for HCC, either hepatectomy or liver transplantation, as the initial treatment. The median time from the diagnosis of HCC to the development of pulmonary metastasis was 30 months (IQR: 10.5–54.0 months). The incidence of pulmonary metastasis followed an inverse exponential pattern, being highest within the first 20 months and decreasing after that (Figure 2).

During the disease course, 17 patients (27.0%) developed intrahepatic recurrence, developing pulmonary metastasis. However, viable HCC was identified in nine patients during the first PM despite repeated local (TACE/TAE) or systemic chemotherapy. In one patient, HCC and concurrent lung metastases were identified during the initial HCC diagnosis. Systemic recurrence occurred before lung metastasis in four patients (adrenal gland in one patient, bone metastasis in three patients), and all were controlled with surgery or remained stable during the first PM.

### 3.2. Pulmonary Metastasectomy

Notably, most of the PMs are performed using a thoracoscopic approach. The extent of surgery was determined based on the location, size, and number of metastatic nodules. Bilateral surgery was performed in 15 patients, left-sided surgery in 25, and right-sided surgery in 23 patients. Notably, most of the metastatic nodules were located in the peripheral region, with only seven (11.1%) in the central region. Pleural adhesions were identified in 13 (20.6%) patients intraoperatively. The number of pulmonary resections (wedge or segmentectomy) performed in each patient was 1.9 ± 1.2. On pathological examination, the mean closest resection margin was 7.85 mm, and the mean maximal size of the metastatic nodules was 16.8 ± 11.4 mm. Wedge resection is the most commonly performed surgical procedure. Additionally, 17 patients (27.0%) underwent procedures other than pulmonary resection. These included nodal sampling or systematic mediastinal lymph node dissection in 12 patients, pericardial mass resection in 1, diaphragm partial resection and repair in 2, and chest wall mass resection in 1 patient. Among the 12 patients who underwent mediastinal lymph node dissection and sampling, metastatic HCC was observed in only one patient, specifically in the 4R node. There were no mortalities or significant morbidities among these patients.

### 3.3. Recurrence after PM

During the follow-up period, intrathoracic recurrence occurred in 45 patients (71.4%). The mean time for intrathoracic recurrence following the initial procedure was 18.5 months, and the recurrence pattern is described in Table 3. Early intrathoracic recurrence within 6 months occurred in 26 patients (57.8%). Repeated PMs were performed in 22 patients (48.9%), a second operation was conducted in 15 (33.3%), and a third operation was performed in 6 patients (13.3%). Local recurrence in the liver was observed in 14 patients. However, other sites of recurrence included the adrenal gland (two patients), spleen (one patient), kidney (one patient), brain (one patient), spine (two patients), pelvic bone (one patient), skull (one patient), and peritoneal seeding (three patients). Overall, 41 patients died during the follow-up period, and 39 of these deaths were attributable to cancer.

### 3.4. Factors Associated with Recurrence-Free Survival and Overall Survival after the First PM

**Figure 3:**
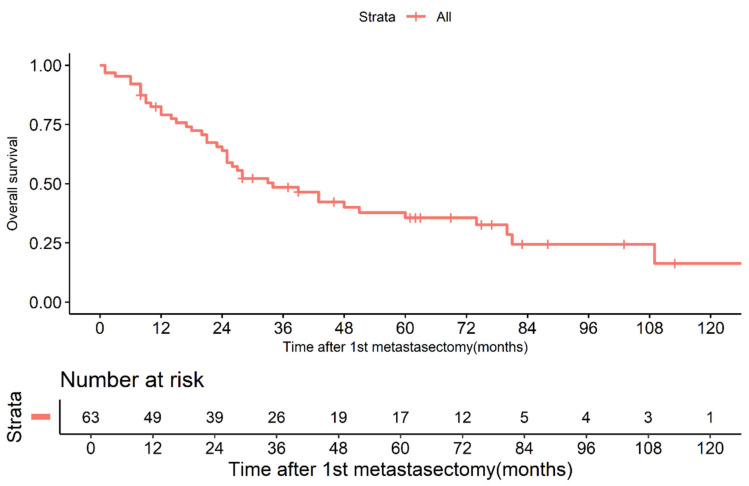
Probability of overall survival in all 63 patients after the first pulmonary metastasectomy.

The cumulative survival rates of the 63 patients following the initial PM at 1, 2, and 5 years were 79.1%, 63.9%, and 35.6%, respectively (Figure 3). The median time to intrathoracic recurrence following the initial PM was 6 months. Therefore, the patients were divided into two groups: those experiencing early (≤6 months) and late recurrence (>6 months).

Univariate and multivariate analyses were performed to identify the factors associated with OS after the first PM. In the univariate analysis, hypertension, alcohol consumption, size of the initial HCC, bilateral PM, number of resected lung specimens, and maximal diameter of the metastatic nodule were associated with the OS (Table 4).

**Figure 4:**
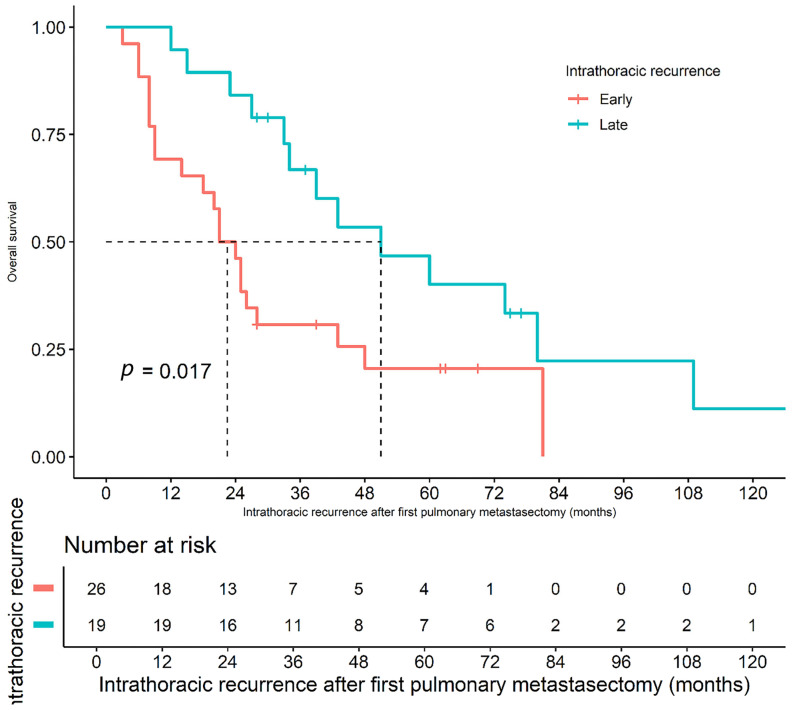
Overall survival based on time to intrathoracic recurrence after first pulmonary metastasectomy (45 patients with recurrence). Median time to recurrence was 6 months; early recurrence (≤6 months) had poorer survival compared to late recurrence (>6 months).

Multivariate models were constructed, and their performance was evaluated using the concordance index and time-dependent receiver operating characteristic analysis, with the final model presented in Table 5. Factors such as early recurrence within 6 months, maximal diameter of the metastatic pulmonary nodule, number of metastatic nodules identified in the pathological specimen, closest margin, and bilateral PM during the first operation were identified as significant predictors of OS. The Harrell C index of the final model was 0.7563, and the area under the curve (AUC) was 85.1%. Figure 4 illustrates the patterns of OS between the early and late recurrence groups. The 1-, 2-, and 5-year survival rates in the early recurrence group were 69.2%, 46.2%, and 20.5%, respectively, whereas the corresponding rates for the late recurrence group were 94.7%, 84.2%, and 40.1%, respectively (log-rank *p* = 0.017).

Early intrathoracic recurrence was identified as a risk factor for poor survival; therefore, an additional comparative analysis was performed to depict the characteristics of the early recurrence group. For the early intrathoracic recurrence group, the initial HCC size was larger (83.10 mm ± 45.5 vs. 52.3 mm ± 45.4, *p* = 0.012), more repeated PM was performed (8 (30.8%) vs. 12 (63.2%), *p* = 0.063), and more lobectomies were performed in the first PM (1.3 ± 0.6 vs. 1.9 ± 0.8, *p* = 0.009) compared with those in the late recurrence group (Table S1).

## 4. Discussion

The prognosis of HCC with extrahepatic metastasis is poor, with a median survival rate of approximately 6–7 months and a 1-year survival rate of 24.9%. Notably, even with partial or complete response to therapy, the 1-year survival rate is <50% [15,16].

The primary treatment for metastatic HCC is systemic; however, the role of surgery in oligometastasis to the lungs in patients with controlled primary intrahepatic HCC remains unexplored. In this study, we evaluated the survival outcomes and predictive factors in patients who underwent PM for HCC. The cumulative 1-, 2-, and 5-year survival rates after PM were 79.1%, 63.9%, and 35.6%, respectively. In the multivariate analysis, early intrathoracic recurrence (≤6 months), the number and size of metastases, resection margin status, and PM bilaterality were significantly associated with OS. A larger size of the primary HCC, higher number of repeated PM, and frequent lobectomy were more prevalent in patients with early (≤6 months) recurrence after PM than in those without early recurrence. Our study showed that PM may provide acceptable survival outcomes in selected patients with smaller lung metastases preferentially confined to the unilateral lung with diseases likely to be resected with a generous surgical margin. Additionally, early recurrence and ensuing reduced OS are expected in patients with a larger initial HCC with prior PM and lobectomy.

The lungs are recognized as the primary and most common locations for metastasis. However, it is difficult to determine the exact incidence of pulmonary metastases in patients with HCC. In a relative incidence study, extrahepatic metastasis was found in 148 of 403 patients with HCCs, with the most common site being the lungs (39%; 58 patients) [17]. Tomimaru et al. found that among 615 patients who underwent radical resection for HCC, pulmonary metastasis was observed in 34 (5.5%) patients during the postoperative follow-up period [18]. In this study, 9,168 patients were diagnosed with HCC and treated at our center between 2004 and 2022, of whom 185 (2.01%) had pathologically proven pulmonary metastasis. This study only included patients with postoperative pathological confirmation; therefore, the incidence could have been higher if we had considered patients who had not undergone surgery.

The generally accepted criteria for PM are not significantly different from those for HCC. PM has been shown to enhance survival in specific subsets of patients, including those whose metastasis is confined to the lungs and those with no residual or recurrent tumors in the liver. However, as Kuo pointed out, the criteria for selecting patients with lung metastasis for PM remain unclear [13,19,20]. Generally, the selection criteria for PM for HCC and other cancers are based on a modification of the principles of Thomford et al. [21]. These include (1) acceptable surgical risk, (2) control of the primary tumor, (3) no evidence of extrahepatic spread, and (4) pulmonary metastases limited to one lung.

However, these criteria have not been applied consistently in recent years. The proportion of reports in which metastasectomy was performed for more than one lesion ranged from 43.8% to 83.5% of the study group [18,22,23,24]. The proportion of bilateral PM performed in the study group ranged from 22.4% to 28.6% [9,13,25]. In addition, synchronous HCC–pulmonary metastasis was reported to be as high as 12.4%, and the PM rate in the presence of other extrahepatic metastases was as high as 17.5% [24]. Therefore, in practice, resectability, lung and liver function, general performance status, and controllability of localized or hyperplastic disease are crucial variables to consider. As Zhou et al. suggested, the indications for surgery for patients with HCC with lung metastases may differ slightly from those for other lung metastases [26].

Previous studies have investigated the potential impact of PM on the overall survival of patients with HCC, focusing on the magnitude of this effect and the subset of patients who may benefit the most. This study demonstrated that the OS rates of the 63 patients following the initial PM at 1, 2, and 5 years were 79.1%, 63.9%, and 35.6%, respectively (shown in Figure 3). In a prognostic study of 20 patients with HCC who underwent PM between 1990 and 2003, the 1- and 3-year survival rates were 45.3% and 23.8%, respectively. Additionally, the recurrence-free survival rates were 32.4% and 21.6% at 1 and 3 years, respectively [27]. Nakagawa et al. reported 1-, 3-, and 5-year survival rates after PM of 80%, 61%, and 36%, respectively, which are consistent with the results of this study [28].

A study comparing pulmonary metastasis after liver transplantation for HCC with and without PM demonstrated a modest survival benefit with PM compared with those without PM (33.5 vs. 10.5 months, *p* = 0.003) [24]. Mizucochi et al. also reported long-term survival with a median survival period of 65 months in a small number of patients with repeated PM after the initial PM; however, this was reported in a small number of patients and should be interpreted cautiously [29]. Considering these disparate retrospective reviews, it can be reasonably inferred that PM demonstrates a survival benefit, at least in patients selected for this treatment.

Furthermore, to analyze the prognostic factors for OS, retrospective analysis was performed, and data were obtained, including age at first PM, sex, size and number of initial HCC, serum alpha-fetoprotein level immediately before PM, recurrence or systemic metastasis of the primary tumor before detection of pulmonary metastasis, characteristics of pulmonary metastases (number, size, and pathologic findings), surgical procedures and approach, and number of PM.

Consequently, several prognostic factors were identified in the final model, including the characteristics of the pulmonary metastasis and the surgical margin, rather than the characteristics of the initial HCC, treatment modality, or stage. In the case of multiple nodules, it was found that the larger the maximal diameter of the largest node, the poorer the OS. Previous studies have indicated that a smaller maximal diameter of ≤3 cm and a smaller number of lesions (three or fewer) are favorable prognostic factors for OS [23,27,30,31]. In this study, bilateral PM was associated with an increased mortality risk, with a hazard ratio (HR) of 8.4 (*p* = 0.001). The authors speculated that the presence of bilateral metastatic nodes may indicate a higher metastatic tumor burden. However, data regarding the prognostic implications of bilateral PM are lacking. Notably, some studies have identified poor prognosis; however, others have not found a relationship between bilateral PM and survival. Therefore, further studies are needed to clarify this association [25,31].

Notably, more metastatic nodules identified during initial surgery reduced the HR to 0.49, suggesting a protective effect against subsequent disease progression. In our study, the number of wedge/segmentectomy resections varied from one to five, with a mean of 1.7, and the number of metastatic nodules detected on pathology ranged from 1.8 to 5, with a mean of 1.5, with a somewhat greater number of metastatic nodules than that in lung resections. These findings prove that removing metastatic nodules, including those identified as suspicious during metastasectomy, is beneficial for OS. However, these results may indicate an increased tumor burden. Therefore, further research is required to better understand the impact of these findings.

The effect of resection margins has been extensively documented. In this study, the HR decreased by 0.93 times for each millimeter increase in the resection margin. Notably, two cases of recurrence were observed in this study, one involving segmentectomy and the other involving wedge resection. The location of a metastatic nodule or multiple metastatic nodules may present technical, anatomical, or practical difficulties in achieving a sufficiently safe margin. Therefore, preserving the normal lung parenchyma is vital. However, it is recommended that a margin of at least 50% of the diameter of the metastatic nodule be maintained as the minimum safety standard [32].

Early intrathoracic recurrence following initial PM was identified as a significant risk factor for OS (HR: 2.7, *p* = 0.009). Therefore, it is essential to interpret studies regarding disease-free intervals cautiously, as the definition of this variable often varies among researchers. However, early recurrence after pulmonary resection is associated with poor prognosis [25]. The researchers sought to identify factors associated with early recurrence but found no significant factors other than the initial HCC size (Table S1).

This study has some limitations. First, the sample size was small. We only studied patients who underwent PM for HCC; therefore, we cannot exclude selection bias because they could have undergone surgical resection. Second, there are issues of long study periods and changes in the medical environment. The study period was relatively long (from 2004 to 2022). The type of initial treatment for HCC did not influence the OS; however, changes in the treatment trends of initial HCC may have influenced pulmonary metastasis and subsequent outcomes (time-dependent confounding).

## 5. Conclusions

In conclusion, aggressive PM may have a favorable effect on OS in patients with sufficient pulmonary and liver functions and controllable extra- and intrahepatic lesions. However, with a sufficient margin, aggressive postoperative surveillance should be considered for patients with large nodules, multiple metastatic nodules, and bilateral PM.

## Figures and Tables

**Table 1:** Definitions of critical clinical parameters used in this study.

Disease-free interval (DFI)	Interval between the first date of pulmonary metastasectomy (T1) and the date of the first occurrence of pulmonary metastasis (T2)
Overall survival (OS)	Interval between the first date of pulmonary metastasectomy (T1) and the date of last follow-up or death (T3)
HCC viability of liver	Presence of arterially enhancing tumor on multiphasic liver CT
Complete remission	Free of any residual primary liver tumor
Intrathoracic recurrence	Recurrence within the intrathoracic cavity, including the lungs, mediastinal lymph nodes, pericardium, parietal pleura, chest wall, and diaphragm, confirmed using serial imaging studies or histopathologically

HCC: hepatocellular carcinoma; CT: computed tomography.

**Table 2:** Clinical characteristics of the 63 patients included in this study.

Variables	Values
Age at first PM (year)	55.5 ± 9.5
Male/female ratio	45/18
Viral status	
HBV	55
HCV	4
None	4
Comorbidities	
DM	7 (11.1%)
Hypertension	10 (15.9%)
Liver cirrhosis	34 (53.9%)
Initial features of liver HCC *	
Size of liver HCC (mm)	68.4 ± 49.15
No. of masses	2.1 ± 2.9
BCLC stage	
0	5
A	24
B	16
C	9
Unknown	9
Initial treatment of HCC	
TACE/TAE	38
RFA	5
Chemotherapy	1
Surgery	19
Serum AFP level at first PM	
<400 (ng/mL)	10
≥400 (ng/mL)	48
Intrathoracic recurrence-free survival from lung surgery (mo) ^†^	18.54 ± 24.55 (median 9.0)
Approach of PM	
Thoracotomy	3
VATS	60
Extent of PM	
Lobectomy	10
Segmentectomy	5
Wedge resection	48
No. of resected lung tissue ^‡^	
1	35
2	9
3	9
4	8
5	2
Maximal pathologic diameter of metastatic nodule (mm)	16.8 ± 11.4
Number of metastatic lesions found on pathology	1.8 ± 1.2
Closest resection margin (mm)	7.85 ± 6.46
Involvement of margin	
Negative	59
Positive	1
Close (<2.0 mm)	3
Laterality of the first PM	
Left	25
Right	23
Bilateral	15
One stage	12
Staged	3
Disease status at the time of the first PM	
Intrahepatic recurrence	17 (27.0%)
Viable HCC	9 (14.3%)
Systematic disease	11 (17.5%)
Other than lung, liver	4 (6.4%)

AFP: alpha-fetoprotein; HBV: hepatitis B virus; HCC: hepatocellular carcinoma; HCV: hepatitis C virus; DM: diabetes; PM: pulmonary metastasectomy; RFA: radiofrequency ablation; TACE: transarterial chemoembolization; TAE: transarterial embolization; VATS: video-assisted thoracic surgery. * Characteristics of liver HCC at the time HCC was first diagnosed. ^†^ Intrathoracic recurrence. ^‡^ The total number of resected lung specimens, either wedge, segmentectomy, or lobectomy.

**Table 3:** Recurrence patterns after the first pulmonary metastasectomy.

	Unilateral PM (*n* = 48)	Bilateral PM (*n* = 15)
Non-recurrence	15	3
Bilateral lung	7	5
Unilateral		6
Ipsilateral	11	-
Resection margin	1	1
Contralateral	12	-
Mediastinum	1	1
Chest wall *	2	0

* Chest wall denotes sites other than the surgical incision site.

**Table 4:** Univariate analysis of overall survival after the first pulmonary metastasectomy.

	HR	95% CI	*p*-Value
Demographics			
Age at first PM (year)	0.997	0.964–1.032	0.871
Male sex	2.406	1.109–5.221	0.026
DM	1.866	0.861–4.045	0.114
HTN	2.321	1.128–4.774	0.022
Liver cirrhosis	1.532	0.826–2.843	0.176
Alcohol	1.856	1.007–3.421	0.048
Smoking	1.557	0.851–2.848	0.151
Characteristics of initial HCC			
Cause of HCC			
Alcohol	1		
Viral	0.518	0.123–2.179	0.369
Unknown	0.298	0.026–3.361	0.328
Initial serum AFP > 400 ng/mL	1.046	0.434–2.521	0.920
No. of initial HCC lesions	1.089	0.992–1.196	0.075
Size of initial HCC (mm)	1.007	1.001–1.014	0.035
Initial treatment of HCC Nonsurgical vs. Surgery	0.917	0.467–1.799	0.800
Clinical presentation at first PM			
High preop serum AFP (>400 ng/mL)	1.046	0.434–2.521	0.920
Viable HCC at the first PM	1.358	0.569–3.239	0.490
HCC recurrence before lung metastasis	0.574	0.265–1.245	0.160
Systematic recurrence before the first PM	0.665	0.306–1.445	0.303
Systemic disease state during PM			
Disease	1		
Free of disease	0.665	0.306–1.445	0.303
Time from HCC diagnosis to pulmonary metastasis	0.996	0.984–1.008	0.504
Characteristics of the first PM			
Laterality of the first PM			
Left: reference	1		
Right	2.003	0.922–4.350	0.079
Bilateral	0.994	0.462–2.137	0.098
Bilateral PM at first			
Same	2.046	1.009–4.151	0.047
Staged	1.827	0.431–7.739	0.413
Bilateral PM at last	1.030	0.557–1.906	0.924
Repeated PM	0.685	0.358–1.313	0.255
Number of total operations	0.683	0.420–1.112	0.125
Sublobar resection	1.542	0.548–4.338	0.411
Additional procedure	0.859	0.420–1.757	0.677
Mediastinal node dissection	1.041	0.459–2.363	0.923
Approach: VATS	1.540	0.365–6.491	0.556
No. of resected lung	1.260	0.964–1.645	0.091
Pathologic findings			
No. of metastatic nodules	1.176	0.911–1.518	0.213
Maximal diameter of metastatic nodule	1.031	1.005–1.057	0.017
Peripheral location of nodule	0.995	0.353–2.794	0.988
Distance of closest margin	0.967	0.908–1.030	0.299
Positive resection margin	1.370	0.419–4.486	0.603
Visceral pleural involvement	1.334	0.182–9.793	0.777

HR: hazard ratio; CI: confidence interval; DM: diabetes mellitus; HTN: hypertension; PM: pulmonary metastasectomy.

**Table 5:** Multivariate analysis of risk factors of overall survival after the first metastasectomy.

Overall Survival	HR	95% CI	*p*-Value
Early recurrence <6 months	2.737	1.280–5.854	0.009
No. of metastatic nodules identified on specimen	0.491	0.282–0.854	0.012
Maximal pathologic diameter of nodule	1.086	1.035–1.140	0.001
Closest margin	0.933	0.872–0.998	0.043
Bilateral PM at first surgery	8.439	2.267–31.413	0.001

CI: confidence interval; HR: hazard ratio; PM: pulmonary metastasectomy; CI: confidence interval.

## Data Availability

All data generated or analyzed during this study are included in this article and its online supplementary material. Further inquiries can be directed to the corresponding author. This study’s dataset is available to the editors, reviewers, and readers upon request from the corresponding author.

## Supplementary Materials

The following supporting information can be downloaded at https://www.mdpi.com/article/10.3390/jcm13144241/s1, Table S1: Characteristics of early intrathoracic recurrence group (≤6 months).
