# Supplementary material for: Prognostic Factors of Pulmonary Metastasectomy for Oligometastatic Hepatocellular Carcinoma Spread to the Lungs
Source: J Clin Med. 2024 Jul 20;13(14):4241. doi: 10.3390/jcm13144241 (PMC11277990; doi:10.3390/jcm13144241)
Supplement: Supplementary file 1 [file jcm-13-04241-s001.zip › jcm-3110341-supplementary.pdf]

Table S1: Characteristics of early intrathoracic recurrence group (≤ 6 months)

	Early (n=26)	Late (n=19)	P value
Age at first PM (year)	54.9 ± 9.82	55.2 ± 7.23	0.892
Male sex	18 (69.2 %)	26 (78.9 %)	0.699
Comorbidities DM	1 (3.8 %)	3 (15.8 %)	0.295
HTN	4 (15.4 %)	3 (15.8 %)	0.999
Liver cirrhosis	13 (50.0 %)	11 (57.9 %)	0.764
Alcohol	16 (61.5 %)	6 (31.6 %)	0.092
Smoking	10 (38.5 %)	4 (21.1 %)	0.330
Viral status			0.747
B	23 (88.5 %0	18 (94.7 %0	
C	1 (3.8 %)	1 (5.3 %)	
N	2 (7.7 %)	0 (0.0 %)	
Initial features of liver HCC*			
Size of liver HCC (mm)	83.10 ± 45.49	52.3 ± 45.4	0.012
No. of mass	2.0 ± 1.94	3.3 ± 4.79	0.255
Initial treatment of HCC			0.501
Chemotherapy	1 (3.8 %)	0 (0.0 %)	
Liver transplantation	1 (3.8 %)	3 (15.8 %)	
RFA	1 (3.8 %)	0 (0.0 %)	
Surgery	6 (23.1 %)	3 (15.8 %)	
TACE/TAE	17 (65.4 %)	13 (68.4 %)	
No. of total PM	1.3 ± 0.55	1.9 ± 0.81	0.009
Repeated PM	8 (30.8 %)	12 (63.2 %)	0.063
Serum AFP level at first PM			0.677
≥ 400 (ng/mL)	5 (21.7 %)	2 (11.8 %)	
< 400 (ng/ml)	18 (78.3 %)	15 (88.2 %)	
Viable HCC at first PM			0.211
Nonviable	20 (76.9 %)	18 (94.7 %)	
Viable	6 (23.1 %)	1 (5.3 %)	
Approach of PM			0.999
Thoracotomy	2 (7.7 %)	1 (5.3 %)	
VATS	24 (92.3 %)	18 (94.7 %)	
Extent of PM			0.310
Lobectomy	2 (7.7 %)	3 (15.8 %)	
Segmentectomy	3 (11.5 %)	0 (0.0 %)	
Wedge	21 (80.8 %)	16 (84.2 %)	
Lymph node dissection	6 (23.1 %)	3 (15.8 %)	0.712
Laterality			0.798
Bilateral	8 (30.8 %)	4 (21.1 %)	
Left	8 (30.8 %)	7 (36.8 %)	
Right	10 (38.5 %)	8 (42.1 %)	
No. of resected lungs	2.3 ± 1.4	1.8 ± 1.1	0.234
No of metastatic nodules on pathologic specimen	2.0 ± 1.4	1.7 ± 1.1	0.449
Maximal pathologic diameter of metastatic nodule (mm)	17.4 ± 12.12	15.4 ± 6.76	0.528
Closest resection margin (mm)	7.4 ± 6.88	9.5 ± 6.37	0.242
Resection margin			0.501

Negative	24 (92.3 %)	19 (100.0 %)
Positive	2 (7.7 %)	0 (0.0 %)

AFP: alpha-fetoprotein; HBV: hepatitis B virus; HCC: hepatocellular carcinoma; HCV: hepatitis C virus; DM: diabetes; HTN, hypertension; PM: pulmonary metastasectomy; RFA: radiofrequency ablation; TACE: transarterial chemoembolization; TAE: transarterial embolization; VATS: video-assisted thoracic surgery. *Characteristics of liver HCC at the time HCC was first diagnosed.
